# SLC4A2 anion exchanger promotes tumour cell malignancy via enhancing net acid efflux across golgi membranes

**DOI:** 10.1007/s00018-021-03890-y

**Published:** 2021-07-19

**Authors:** Elham Khosrowabadi, Antti Rivinoja, Maija Risteli, Anne Tuomisto, Tuula Salo, Markus J. Mäkinen, Sakari Kellokumpu

**Affiliations:** 1 Faculty of Biochemistry and Molecular Medicine, University of Oulu (Oulun Yliopisto), Aapistie 7A, PO BOX 5400, 90014 Oulu, Finland; 2 Present Address: Northern Finland Laboratory Centre, Oulu, Finland; 3 Cancer and Translational Medicine Research Unit, University of Oulu, Oulu, Finland; 4 Medical Research Centre, Oulu University Hospital, Oulu, Finland

**Keywords:** Cancer, Invasion, Golgi apparatus, Glycosylation, Proton leak, Net acid efflux homeostasis

## Abstract

Proper functioning of each secretory and endocytic compartment relies on its unique pH micro-environment that is known to be dictated by the rates of V-ATPase-mediated H^+^ pumping and its leakage back to the cytoplasm via an elusive “H^+^ leak” pathway. Here, we show that this proton leak across Golgi membranes is mediated by the AE2a (SLC4A2a)-mediated bicarbonate-chloride exchange, as it is strictly dependent on bicarbonate import (in exchange for chloride export) and the expression level of the Golgi-localized AE2a anion exchanger. In the acidic Golgi lumen, imported bicarbonate anions and protons then facilitate a common buffering reaction that yields carbon dioxide and water before their egress back to the cytoplasm via diffusion or water channels. The flattened morphology of the Golgi cisternae helps this process, as their high surface-volume ratio is optimal for water and gas exchange. Interestingly, this net acid efflux pathway is often upregulated in cancers and established cancer cell lines, and responsible for their markedly elevated Golgi resting pH and attenuated glycosylation potential. Accordingly, AE2 knockdown in SW-48 colorectal cancer cells was able to restore these two phenomena, and at the same time, reverse their invasive and anchorage-independent growth phenotype. These findings suggest a possibility to return malignant cells to a benign state by restoring Golgi resting pH.

**Supplementary Information:**

The online version contains supplementary material available at 10.1007/s00018-021-03890-y.

## Introduction

Altered cellular metabolism, tumour acidosis, and aberrant glycosylation are all hallmarks of cancers and contribute to tumorigenesis and its progression by various means [1–4] Cancer-associated glycosylation changes most often include increased branching and/or fucosylation of N-linked glycans, synthesis of truncated mucin-type O-glycans, increased sialylation and decreased sulphation of glycosaminoglycans such as heparan sulphate [5]. Because cell-surface glycans regulate a vast number of different cell–cell and cell–matrix interactions [6, 7], their alterations can modulate a variety of basic cellular functions, including inflammatory responses, immune evasion, apoptosis, cell attachment as well as cancer cell dissemination, motility, invasion, and metastasis [8–12].

Despite most cell surface glycans (collectively known as the glycocalyx) are made in the Golgi apparatus by the consecutive actions of Golgi-resident glycosyltransferases, the mechanistic details behind cancer-associated glycosylation changes are incompletely understood. Potential causes include altered expression of glycosyltransferases, their mislocalization, loss of catalytic activity, and inability to form functionally relevant complexes in the Golgi [13, 14]. Moreover, previous work from our laboratory suggests that Golgi resting pH in cancer cells is abnormally high [15] and that pH gradient dissipating compounds (chloroquine, ammonium chloride) can induce cancer-associated glycosylation changes in normal cells and lead to mistargeting of apical glycoproteins in epithelial cells [16–18].

Normally, Golgi acidity increases along the cis–trans axis from pH 6.7 (cis-Golgi) to pH 6.5 (medial-Golgi), reaching pH 6.3 at the trans-Golgi and pH 6.0 in the trans-Golgi network, TGN [19, 20]. It is set mainly by three main ion transport systems: the vacuolar (V)-ATPase (that pumps protons to Golgi lumen), a chloride channel (the GPHR or the GOLAC) that by importing Cl^−^ prevent a build-up of membrane potential brought about otherwise by proton pumping, and a proton leak “channel” that allows the escape of protons back to the cytoplasm across Golgi membranes [21–24]. Of these, the proton leak pathway may be the most important one for setting organelle resting pH or “set point”, because its rate has been shown to decrease along the secretory pathway (ER, Golgi, secretory vesicles) concomitantly with their resting pH [23].

While both the V-ATPase and the GPHR protein are well characterized at the molecular level, the identity of the net acid efflux pathway remains an enigma. Previous physiological measurements have suggested that net acid (proton) efflux across Golgi membranes is voltage-sensitive and inhibited by Zn^2+^, suggesting the involvement of a regulated “channel” [24]. Other studies have proposed that proton leak involves NHE7 and NHE8 Na^+^/H^+^ exchangers [25]. However, a recent study shows that these exchangers act as acid loaders, and not as acid extruders, in the Golgi [26, 27]. The AE2a (SLC4A2a) bicarbonate-chloride exchanger variant that is expressed in the Golgi membranes in a variety of cell types [28], could also contribute to Golgi resting pH either by importing or exporting HCO_3_^−^ to or from the Golgi lumen. In support of this, all the members of this Na^+^-independent Cl^−/^HCO_3_^−^ exchanger gene family (SLC4A1-4 or AE1–AE4) are known to mediate a diisothio-cyanatostilbene-2,2-disulfonate (DIDS)-sensitive, electroneutral and obligatory one-to-one exchange of chloride for bicarbonate [29, 30]. Thereby, they can regulate intracellular pH (pHi), cell volume, and chloride concentration in the cells. Of these, the AE2 isoform is detected in nearly all tissues and cells examined, and therefore, is said to have a “housekeeping” role in the cells. Consistent with this, AE2 knockout in mice causes a severe phenotype, as AE2^−/−^ mice often die either before or at weaning [31].

AE2 has at least 3 known variant polypeptides (AE2a-c) which differ from each other by their variable N-terminal domains [29, 30]. Both the N- and C-termini of the AE2 variant polypeptides are known to be cytoplasmic and can interact with proteins that regulate either the activity or its subcellular localization in the cells. The cytoplasmic C-terminal tail in turn has been shown to interact with carbonic anhydrase II (or IV), an enzyme in the cytoplasm that produces bicarbonate anions (and protons) for transport [32, 33]. This interaction was found to enhance AE anion transport activity as well as the catalytic activity of the CAII [34], suggesting that they form a “transport metabolon”. However, its existence was later challenged [35], and therefore needs to be formally verified. The N-terminus of the AE2a (the longest N-terminal variant) in turn has been shown to bind ANK195, a Golgi-specific ankyrin isoform [36] that links the AE2-ANK195 complex to βIII spectrin-based Golgi membrane skeleton [37]. Thus, this interaction likely is responsible for the localization of the AE2a variant in the Golgi.

To find out whether the AE2a variant, indeed, contributes to Golgi resting pH, we measured directly its effect on Golgi resting pH in COS-7 cells that express an endogenous AE2a variant in the Golgi [28, 36], in cells that overexpress it, or in cells in which the AE2a variant was knocked down with AE2-specific shRNAs. Previously, we have shown that COS-7 cells do not express other AE2 variants at a detectable level in the Golgi or the plasma membrane [28]. By using ratiometric and Golgi-targeted pHluorin as the probe [38], we now show that the AE2a variant regulates Golgi resting pH by facilitating net acid efflux across Golgi membranes via the well-known chemical buffering reaction that yields carbon dioxide and water from luminal bicarbonate anions and protons. The functional relevance of this pathway was also verified by showing that it is often upregulated in cancers and established cancer cell lines, consistent with their elevated Golgi resting pH and altered glycosylation status. Intriguingly, we also show that AE2 knockdown in one of these cell lines (SW-48) was not only able to restore an acidic Golgi resting pH and its glycosylation potential, but also to reverse their invasive and anchorage-independent growth phenotype. Our lectin microarray glycan profiling also uncovered two potential glycotopes that may govern this phenotypic change between the cells.

## Results

### AE2a expression level alters Golgi resting pH and its glycosylation potential

**Fig. 1:**
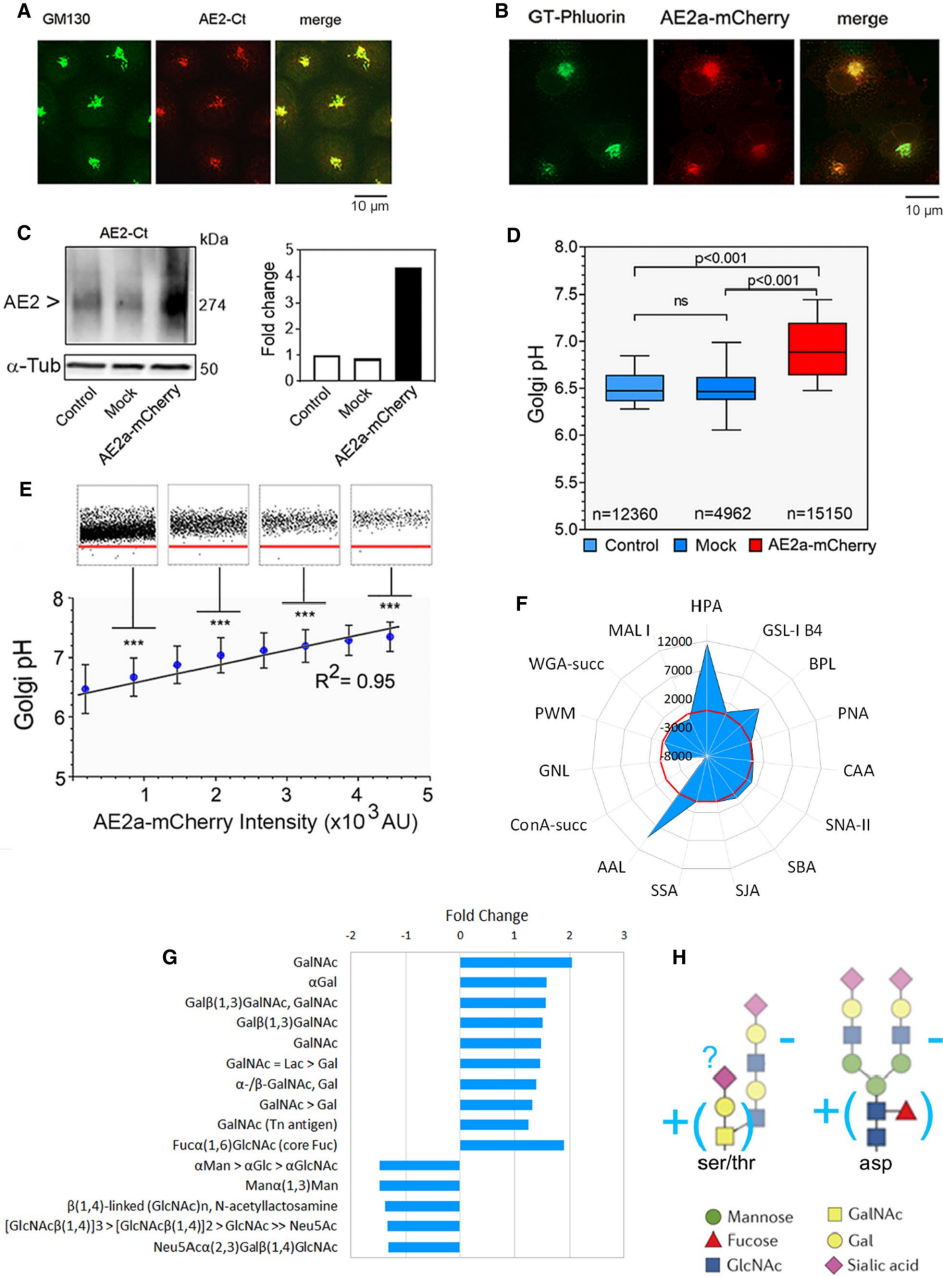
AE2a overexpression in COS-7 cells alters Golgi resting pH and its glycosylation potential. **A** Immunofluorescence microscopy of the endogenous AE2a protein and its co-localization with the Golgi marker (anti-GM130) in COS-7 cells. **B** Fluorescence microscopy of COS-7 cells transfected with the GT-pHluorin- and AE2a-mCherry-encoding plasmids. The merged figure shows their co-localization in the Golgi. **C** Quantification of the AE2a-mCherry protein level in wild-type COS-7 cells (control), in mock-transfected cells, and AE2a-mCherry expressing cells. The protein was visualized after Blue Native-PAGE (BN-PAGE) by immuno-blotting with the anti-AE2Ct antibody. **D** Ratiometric Golgi pH measurements in wild type, mock-transfected, or AE2a-mCherry (red) expressing COS-7 cells. AE2a-mCherry overexpressing cells were selected using the Operetta TM build-in software using the mCherry tag as a marker. In the box blot analysis, the whiskers indicate 10th to 90th percentiles; *n* = number of cells used for analyses. **E** Regression analysis between Golgi resting pH and AE2a-mCherry expression level. Single-cell data points (dots) were classified into eight equal classes based on AE2a-mCherry intensity (200–5000 AU units, 600 AU units/class), and plotted against the Golgi resting pH. Single-cell data points are shown for every second class. The red line (pH 6.0) is added to help comparison. The means (± SD, *n* > 2000 cells) were used to obtain the fitted regression line and its coefficient (*R*^2^ = 0.95). **F** Lectin binding differences between AE2a-mCherry overexpressing COS-7 cells and mock-transfected control cells. Total binding intensities (Fig S1C) were used to calculate the means (± SD, *n* = 6) to select statistically significant (*p* < 0.05) changes between the cells. A two-tailed student’s *t *test was used for statistical analysis. The means were then subtracted from each other (AE2a-mCherry minus control) to get the subtracted fingerprint shown as a radar plot. The red-colored ring denotes zero lines (no difference). **G** A bar graph showing significant differences (**F**) as fold changes. The glycotopes specific for each lectin are listed in a clockwise manner starting from the HPA lectin. **H** A cartoon that illustrates main N- and O-glycan differences between AE2a-mCherry overexpressing cells and mock-transfected controls

We chose COS-7 cells as target cells mainly because they endogenously express the AE2a variant in the Golgi and do not express other AE2 transcripts or variant proteins at detectable levels at the plasma membrane [28, 36]. The cells also display a normally acidic (pH 6.3–6.5) Golgi resting pH [18]. We confirmed this by both immunofluorescence (Fig. 1A) and immunogold cryo-electron microscopy (Fig. S1A). Staining of COS-7 cells with an affinity-purified anti-C-terminal AE2-antibody that recognizes all the AE2 variants confirmed an almost exclusive localization of the AE2a in the medial/trans-Golgi cisternae and the trans-Golgi network (TGN).

To investigate whether the AE2a variant regulates Golgi resting pH, we determined Golgi resting pH in live wild-type COS-7 cells and in cells that overexpress the same variant. By using the medial/trans-Golgi-targeted pHluorin (GT-pHluorin, Fig. 1B) as a probe. Ratiometric measurements showed that AE2a overexpression increased significantly (*p* < 0.001) Golgi resting pH by 0.5 pH units (from pH 6.4 to 6.9; Fig. 1D) when compared to wild type or mock-transfected controls (Fig. 1C). Single-cell data analyses also revealed a strict correlation (*R*^2^ = 0.95) between the AE2a expression level and the Golgi resting pH (Fig. 1E).

Since Golgi acidity is known to be important for proper glycosylation [17, 18], we next assessed how AE2a overexpression alters the COS-7 cell glycosylation profile. Lectin microarray glycan profiling (Fig. S1B) showed that out of the 43 lectins, 15 showed significant (*p* < 0.05) changes in their binding to their relevant glycotopes (Fig. 1F). The main changes included upregulation of truncated O-glycan glycotopes GalNAc (the Tn-antigen) and Galβ (1,3)-GalNAc (the T-antigen) as well as core-fucosylated N-glycans (Fig. 1G). Other N-glycan recognizing lectins, instead, showed downregulation of terminal mannose-, *N*-acetylglucosamine-, galactose- and α(2,3)-linked sialic acid-containing N-glycans. Collectively, these findings suggest that the elevated Golgi resting pH in AE2a overexpressing cells impairs glycosylation either by enhancing the synthesis of O-glycan and N-glycan “core” structures or by attenuating their elongation (Fig. 1H). Since they are normally masked by further glycosylation in the Golgi [4, 5, 7, 12], the latter possibility is more likely.

**Fig. 2:**
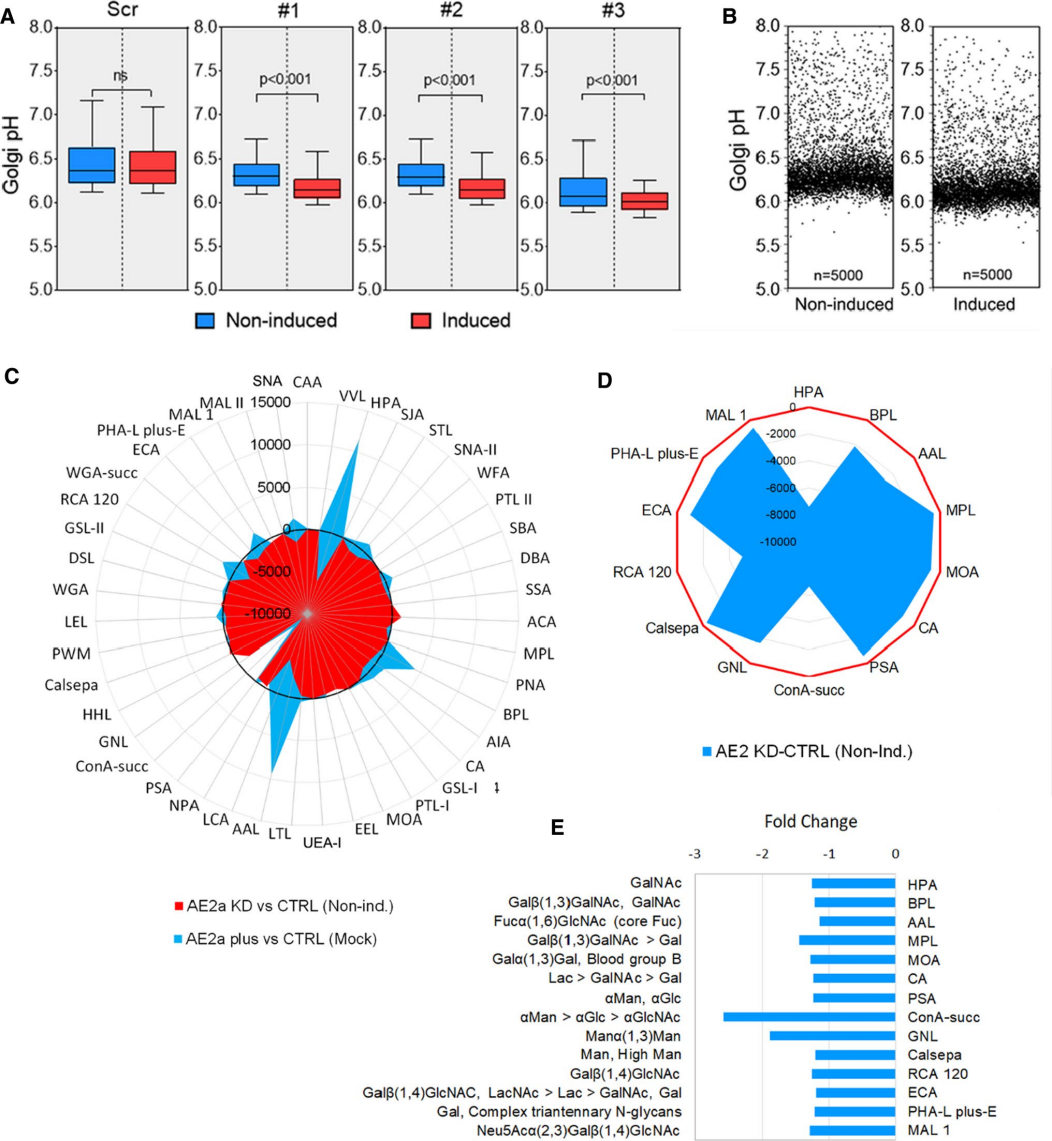
AE2a knockdown decreases Golgi resting pH and alters glycosylation. **A** Golgi resting pH in AE2a knockdown cells. Stably AE2 shRNA expressing COS-7 cells were transfected with the GT-pHluorin plasmid, and either induced (red boxes) or not (blue boxes) before determining their Golgi resting pH by ratiometric imaging (*n* = 5000 cells/group). The whiskers above and below the box indicate 10th to 90th percentiles. **B** Single-cell analysis of the COS-7 cell Golgi resting pH in AE2a knockdown cells carrying the shRNA#1 construct. The Golgi resting pH of both non-induced and induced cells is shown. **C** Comparison of the lectin binding differences between AE2a overexpressing and knockdown cells. The subtracted fingerprints shown were obtained by calculating the difference between their total lectin binding intensities and control cells (mock-transfected and non-induced cells, respectively). The total binding intensities (Fig. S1C-D) were used for the calculations. **D** Subtracted fingerprint showing significant (*p* < 0.05) glycosylation differences between AE2a knockdown (induced) and control (non-induced) cells. Significant differences were calculated as above and are shown as a radar plot. The red circle denotes zero values (no difference). **D** A bar graph showing significant differences (**C**) as fold changes. The corresponding glycotopes to lectins shown in **C** are shown clockwise starting from HPA lectin

Knockdown of the endogenous AE2a protein in COS-7 cells with AE2-specific shRNAs provided further support for its role in Golgi pH regulation. For knockdown, we used a doxycycline inducible SMART™ vector system, because it allows adjustment of the knockdown efficiency and thus, preservation of cell viability, in contrast to its complete knockout in mice [31]. In stably transfected cells, all three AE2-specific shRNAs used (Table S1) diminished the level of the AE2a protein by 10–60% depending on the Doxycycline concentration used (Fig. S1C). They also decreased Golgi resting pH by ~ 0.2 pH units in induced cells, when compared to non-induced controls or wild type or scrambled shRNA with or without the induction (Fig. 2A). Single-cell data analyses in shRNA#1 knockdown cells also confirmed that the Golgi resting pH was decreased uniformly in most cells of this cell population (Fig. 2B).

### AE2a knockdown also altered COS-7 cell glycosylation potential

Lectin microarray glycan profiles (Fig. S1D) showed that these changes, in general, were opposite to those detected upon AE2a overexpression (Fig. 2C). Only two lectins (succinylated ConA and GNL) displayed significantly lower binding of mannose-type N-glycans in both AE2a knockdown and AE2a overexpressing cells. Other lectins (Fig. 2D) also displayed significantly diminished binding of their specific glycotopes. The fold changes, however, did not exceed more than 1.5-fold (Fig. 2E), except in the case of the two mannose-type N-glycan specific lectins (succinylated ConA and GNL) which exhibited roughly twofold lower binding of their glycotopes (Fig. 2E). These findings indicated that not only AE2a overexpression but also its knockdown in COS-7 cells, slightly impairs glycosylation of N-and O-glycans, reflecting their opposite effects on Golgi pH and its extent. The main differences detected between these two cell types suggest that AE2a knockdown may specifically inhibit O-glycan synthesis and N-glycan branching, while AE2a overexpression affects their elongation. More detailed glycan analyses are needed, however, to clarify this issue.

### AE2a-mediated bicarbonate import enhances net acid efflux across the Golgi membranes

**Fig. 3:**
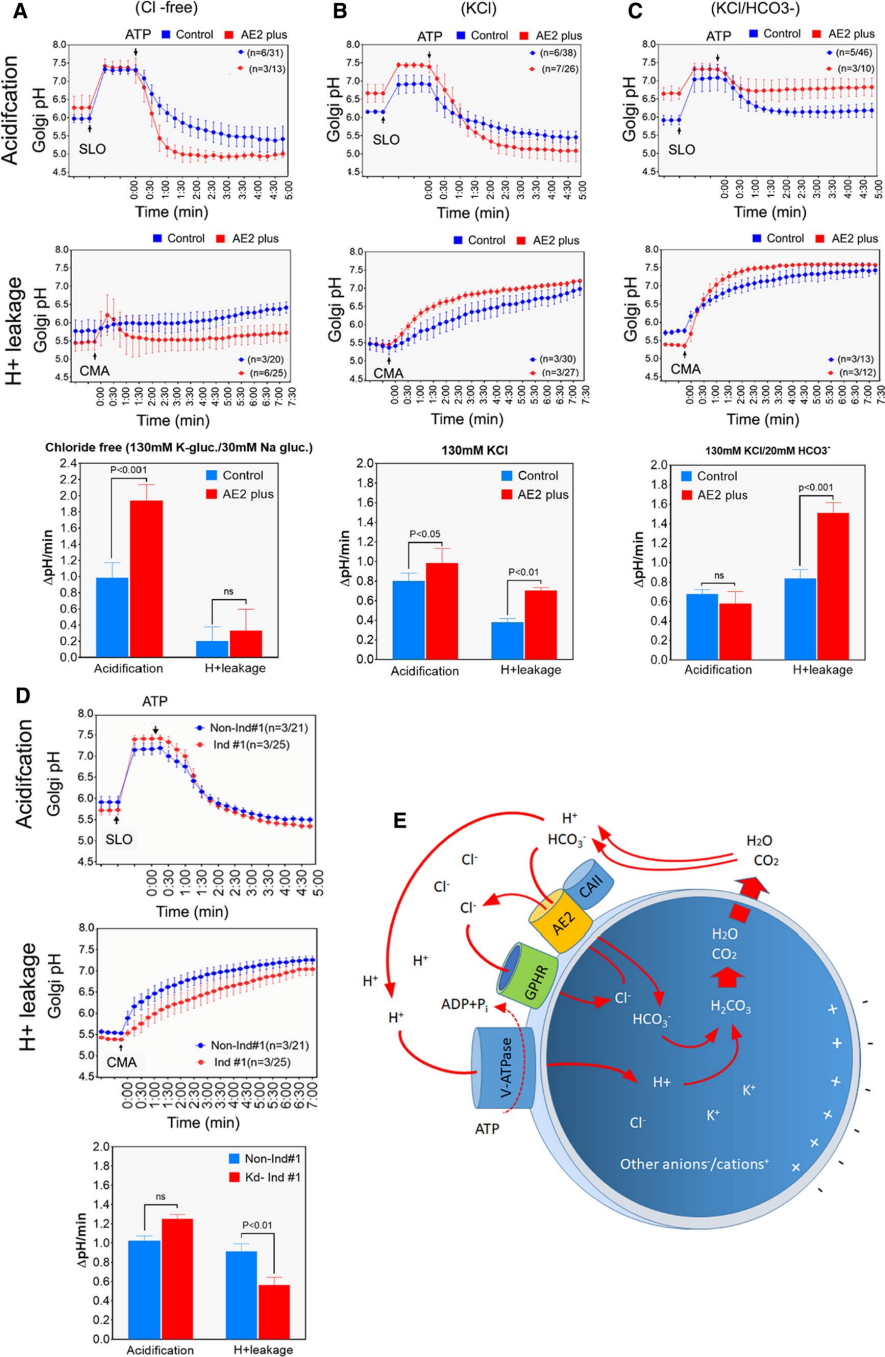
AE2a alters Golgi resting pH via facilitating net acid efflux across Golgi membranes. Cells transfected with GT-pHluorin plasmid, either with or without the AE2a-mCherry plasmid, were permeabilized with SLO before ratio-imaging of the cells. Cells were kept in different bath solutions during the experiment: **A** no Cl^−^ nor HCO_3_^−^, **B** only Cl^−^ (no HCO_3_^−^) and **C** both Cl^−^ and HCO_3_^−^. Arrows denote the addition of SLO, ATP, or CMA to the bath medium. The obtained ratios were transformed to pH values (mean ± SD, n ≥ 3/total number of cells used in the experiments) using an equation obtained from the calibration standard curve. The pH values were then plotted against time. The bars (bottom panels) depict the initial acidification and leakage rates (∆pH/min) that were calculated from the fitted curves in each case. **D** Golgi acidification and net acid efflux rates in AE2 knockdown (induced) COS-7 cells and in control cells (non-induced) carrying the same plasmid (shRNA#1). Golgi acidification rate measurements were done in the absence (top) of 20 mM bicarbonate to be able to compare the maximal proton pumping rates by the V-ATPase. Proton leak rate measurements in control and AE2 KD cells. The pH values as well as the initial Golgi acidification and net acid efflux rates (ΔpH/min) were obtained as above and plotted against time. 20 mM bicarbonate was present in the bath solution to allow maximal net acid efflux rate from the Golgi lumen. **E** The deduced model describing the AE2a-mediated net acid efflux pathway across the Golgi membranes. The main ions as well as their corresponding transporters are shown

Next, we carried out Golgi acidification and proton-leakage rate measurements in COS-7 cells to assess why AE2a overexpression increases Golgi resting pH, and knockdown decreases it. To accomplish this, both control cells carrying an empty vector (mock-transfected cells) or cells overexpressing the AE2a variant (AE2plus cells) were transfected with the GT-pHluorin plasmid. One day later, the plasma membrane of the cells was first permeabilized with streptolysin O (SLO) [39] to allow controlled removal or addition of ATP, Cl^−^ or HCO_3_^−^ to the cytoplasm. Golgi pH changes were then followed by ratiometric imaging of the selected Golgi regions. In Cl^−^ /HCO_3_^−^ free bath medium (to minimize AE2a anion exchange activity), the addition of excess ATP (10 mM) resulted in marked acidification of the Golgi lumen (Fig. 3A top panel) before it plateaued at pH 5.0–5.5 both in AE2a overexpressing and mock-transfected control cells, respectively. The calculated initial acidification rates (ΔpH/min) were 1.0 and 1.9 pH units/min (Fig. 3A bottom panel), i.e. significantly higher in AE2 plus cells than in control cells. Proton “leakage” or efflux rates measured after blocking the V-ATPase with 1 µM Concanamycin A (CMA) further showed that they were low, and the initial leakage rates did not exceed 0.35 pH units/min in either case (Fig. 3A, middle and bottom panels).

When Cl^−^ anions were added to the bath medium (by replacing KCl with K-gluconate), Golgi acidification rate in AE2a overexpressing cells was still significantly higher than that of control cells (0.8 vs. 1.0 pH units/min, (Fig. 3B top and bottom panels), although Cl^−^ addition decreased the initial ΔpH/min from 1.9 ΔpH/min (no chloride) to 1.2 ΔpH/min (with chloride, Fig. 3B bottom panel). In contrast, the presence of Cl^−^ in the bath medium increased net acid efflux rates (Fig. 3B, middle panel) in both control and AE2a overexpressing cells by ~ twofold when compared to the rates in Cl^−^ -free bath medium (Fig. 3B, bottom panel), probably due to endogenous activity of carbonic anhydrase II (CA II) and the production of HCO_3_^−^. In addition, the H^+^ leakage rate was significantly higher in AE2a overexpressing cells than in mock-transfected control cells (Fig. 3B, middle and bottom panels).

Addition of Cl^−^ and HCO_3_^−^ to the bat medium slowed down Golgi acidification in both control and AE2a overexpressing cells (Fig. 3C). Golgi luminal pH also plateaued at pH 6.3 (control cells) or pH 6.7 (AE2a overexpressing cells). These changes coincided with markedly faster net acid efflux rates in both cell types (Fig. 3C middle and bottom panels). AE2a overexpressing cells also displayed a significantly higher leak rate than mock-transfected control cells, suggesting that net acid efflux across Golgi membranes is strictly dependent on the presence of chloride and bicarbonate anions in the bath media as well as the expression level of the AE2a protein in the Golgi membranes of the cells. In support of this view, AE2a knockdown cells displayed a significantly lower net acid efflux rate than did non-induced control cells (Fig. 3D, middle and bottom panels) while their Golgi acidification rate was not affected. In addition, when cells were bathed in different bicarbonate-free buffer solutions (containing SLO and adjusted to pH 6, pH 7, pH 8, respectively), we did not detect marked differences in the Golgi resting pH between the cells (Fig. S2A). On the other hand, cultivation of intact COS-7 and HeLa cells in 40 mM bicarbonate (normally 20 mM) increased significantly (*p* < 0.001) Golgi resting pH in the cells (Fig. S2B).

To evaluate whether the model is feasible also electrochemically, we calculated electrochemical driving forces for AE2a-mediated chloride-bicarbonate exchange. Thus, the V-ATPase generated H^+^ gradient (~ 1 pH unit) across Golgi membrane creates a + 61.5 mV Nernst potential that at pH_G_ 6.0 and pH_i_ 7.0 corresponds to -61.5 mV electrochemical driving force for either influx of negative ions (Cl^−^) or efflux of positive ions (K^+^, Na^+^) or both. These ion fluxes will then counteract to balance the “proton motive force”, consistent with the notion that membrane potential across Golgi membranes is non-existent [24]. At equilibrium (107 mM [K^+^]_G_; 140 mM [K^+^]_i_, [24]), Nernst equation calculations indicated that K^+^ efflux would equal an equilibrium potential of -7 mV. Assuming that Cl^−^ influx will balance the rest (−54 mV) of the “H^+^ motive force”, [Cl−]_G_ would equal to ~ 0.5 mM when [Cl^−^]_i_ is set to 4 mM [43]. Due to continuous proton pumping [24] and the consumption of HCO_3_^−^ anions for Golgi buffering, its concentration in the Golgi lumen will remain low, compared to its concentration in the cytoplasm (12 mM HCO_3_^−^, [43]). Therefore, a high [HCO_3_^−^] gradient between the cytoplasm and the Golgi is expected to favour AE2a-mediated bicarbonate import in exchange for chloride export against its smaller concentration gradient across Golgi membranes, in accord with the model.

Based on these observations, we suggest a model (Fig. 3E and supplementary video) in which AE2a-mediated bicarbonate import (in exchange for luminal chloride) facilitates net acid efflux across Golgi membranes via the well-known bicarbonate buffering reaction (H^+^  + HCO_3_^−^ > H_2_CO_3_ > H_2_O + CO_2_, [40]) that produces carbon dioxide and water from luminal bicarbonate anions and protons before their egress to the cytoplasm via diffusion or aquaporin water channels [41, 42]. Their egress is likely helped by the flattened shape of the Golgi cisternae, as their high surface-to-volume ratio (as in erythrocytes) is optimal for water and gas exchange. It is perhaps also not a mere coincidence that the pKa 6.4 of the bicarbonate-carbonic acid reaction is within the same range as the Golgi resting pH (pH 6.7–6.0).

### AE2 mRNA and protein levels are often upregulated in cancers and cancer cell lines

The Cancer Genome Atlas mRNA expression database (matched TCGA and GTEx data) analyses suggested that the AE2-mediated net acid efflux pathway may have functional relevance in cancer cells because the AE2 mRNA is often upregulated in cancer tissues compared to matched normal tissues. Because the mRNA nor the AE2_CT_ antibody analyses do not discriminate between different AE2 variants (AE2a-c), the term “AE2” will be used from here onwards. Interestingly, mRNA level analyses showed significantly (*p* < 0.05) upregulated AE2 mRNA levels in more than half (16/31) of the cancer types present in the database. Of these, six cancer types showed highly significant (*p* < 0.001) upregulation of the AE2 mRNA (Fig. S3A). Kaplan–Meier survival plots also suggested that a high AE2 mRNA expression level increases death risk by 1.6-fold (Fig. S3B). In addition to these six cancer types, analyses using only the matched TCGA as controls revealed that the AE2 mRNA is also significantly (*p* < 0.05) upregulated in colon and rectum carcinomas (Fig. S3C). Survival plots also indicated 1.3-fold and 5.4-fold increased death risk in these patients, respectively (Fig. S3D, E). Immunoblotting of AE2 protein in seven low-grade colorectal cancer tissue specimens further verified its variable expression in cancer tissues. We found that four (57%) displayed more than 1.5-fold higher AE2 protein levels than the control tissue specimens (Fig. S3F). In addition, immunostaining of colorectal cancer tissue sections revealed that the AE2 protein is present in the Golgi membranes but also at the plasma membrane of cancer cells (Fig. S3G). Taken together, the above in vivo findings suggested that AE2 overexpression in the Golgi and/or the plasma membrane may contribute to tumorigenesis and/or its progression at least in some cancer cell types.

### AE2 overexpression in SW-48 cells is responsible for their elevated Golgi resting pH

**Fig. 4:**
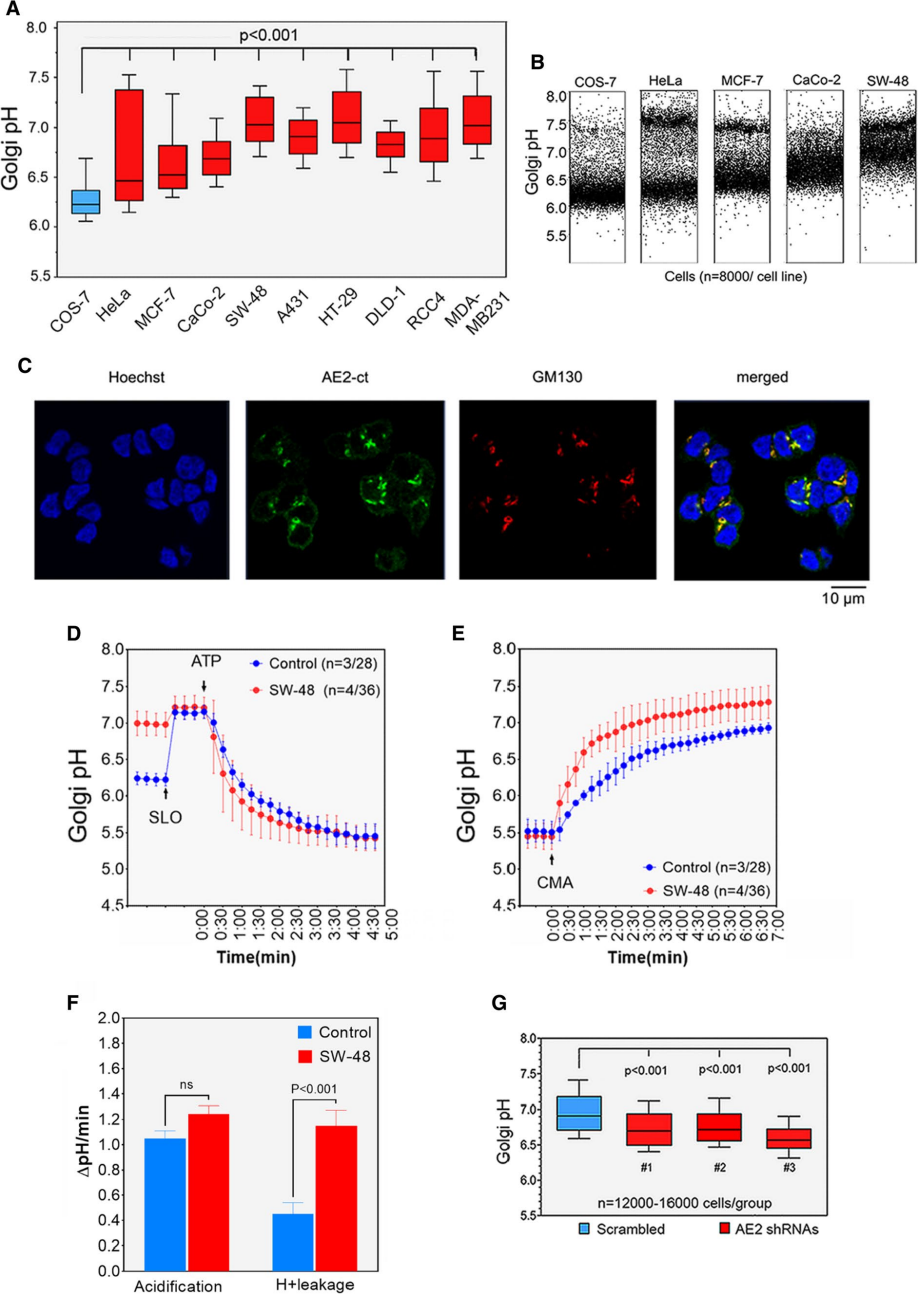
AE2 upregulation in cancer cells is responsible for their elevated Golgi resting pH. **A** Golgi resting pH in different cancer cell types in comparison to non-malignant control cells. Golgi resting pH in each cell type was determined as above (Fig. 1) using the GT-pHluorin as a probe. The values are shown using box plots. Whiskers above and below each box indicate 10th to 90th percentiles. **B** Single-cell analysis of the Golgi resting pH in the four depicted cancer cell types and COS-7 cells. Golgi pH values from 8000 cells in each case were blotted individually for each cell type. Note the two distinct cell populations which differ in the Golgi resting pH in three cell lines. **C** Immunofluorescence microscopy of the endogenous AE2 protein showing its localization in the Golgi and at the plasma membrane (faint circle outside the nucleus) of SW-48 cells. Fixed cells were co-stained with the anti-AE2Ct antibody and with a Golgi marker antibody (GM130). Golgi acidification (**D**) and net acid efflux (**E**) rates in SW-48 cells. The experimental set-up is identical to that described in Fig. 3. The calculated initial acidification and leakage rates (ΔpH/min) are also shown (**F**). **G** Golgi resting pH in SW-48 cells after AE2 knockdown. Stably transfected SW-48 cells carrying either the shRNA#3 or the control plasmid (scrambled shRNA) were induced for 3 days using 100 ng/ml of Doxycycline. Golgi resting pH values were then determined as above. The whiskers above and below the box indicate 10th to 90th percentiles

Immunoblotting of the AE2 protein in different cancer cell lines revealed further that the protein was also overexpressed in four out of the nine (44%) cell lines tested (Fig. 3H). These four AE2 overexpressing cell lines (SW-48, HT-29, RCC4, and MDA-MB231) also displayed the highest Golgi resting pH values that ranged between pH 6.9–7.2 (Fig. 4A). Intriguingly, however, Golgi resting pH was also elevated (pH_G_ 6.5–6.8) in all the other cancer cell lines as well, even though they did not overexpress the AE2 protein. Statistical analyses also failed to reveal a correlation between the AE2 expression level and the Golgi resting pH in the cell types used (*R* = 0.59, *p* > 0.05). Therefore, we anticipate that in cell types that express the AE2 protein at comparable or lower levels than COS-7 cells, changes in cytoplasmic pH or the activity of enzymes or other ion transporters contribute to Golgi resting pH, are likely responsible for their elevated Golgi resting pH. In addition, single-cell data analyses (Fig. 4B) demonstrated that HeLa and MCF-7 cells, in particular, display two distinct cell populations that differ in their Golgi resting pH. Their co-existence was not associated with poor cell survival or cell death, as such cells do not resist repeated washings and media changes during the experiments. Golgi fragmentation that is typical in cancer cells (Fig. S4A) was also not the reason, as both compact and fragmented Golgi elements had rather similar Golgi resting pH values (Fig. S4B). Moreover, despite elevated Golgi resting pH, HeLa cells have a compact Golgi (Fig. S4A and S4C). Instead, the co-existence of the two cell populations likely reflects adaptation to hypoxia, as exposure of MCF-7 and SW-48 cells to hypoxia (5% O_2_ instead of 16% O_2_) for 48 h decreased Golgi resting pH only in “high pH” cell population while it did not affect the “low pH” cell population (Fig. S4D).

To assess the role of the AE2 overexpression in more detail on tumorigenesis, we chose SW-48 colorectal cancer cells as our targets mainly because nearly all (98%) SW-48 cells displayed markedly elevated Golgi resting pH (Fig. S4E). Most of them also bind PNA lectin (Fig. S4F), a sign of altered glycosylation. The cells were also found to contain comparable levels of ATP to COS-7 cells (Fig. S3G, H) which should sustain proton pumping if the V-ATPase is functional in the cells. The cells also express a substantial amount of the AE2 protein in the Golgi membranes, yet the protein is detectable also at the cell surface (Fig. 4C), in line with our in vivo results (Fig. S3G). Golgi acidification and net acid efflux rate measurements in the cells revealed that the Golgi lumen was acidified at a comparable rate and extent to COS-7 cells (Fig. 4D), indicating that the V-ATPase is fully functional and therefore not responsible for their elevated Golgi resting pH. In contrast, the net acid efflux rate (Fig. 4E, F) was significantly (by 2.6-fold) higher than that of COS-7 cells (ΔpH 1.15 pH units/min vs. ΔpH 0.45 pH units/min, respectively). In addition, AE2 knockdown in the cells (Fig. S5A) decreased significantly their Golgi resting pH from pH 6.9 (Scr) to 6.6 (Fig. 4G). It also decreased significantly their PNA lectin binding activity (Fig. S5B) as well as the percentage of PNA-positive cells in the cell population (Fig. S5C). AE2 knockdown also slightly elevated cytoplasmic resting pH in the cells (Fig. S5D), consistent with its functioning against alkaline loads [29, 30].

### AE2 knockdown in SW-48 cells reverses their invasive and anchorage-independent growth phenotype

**Fig. 5:**
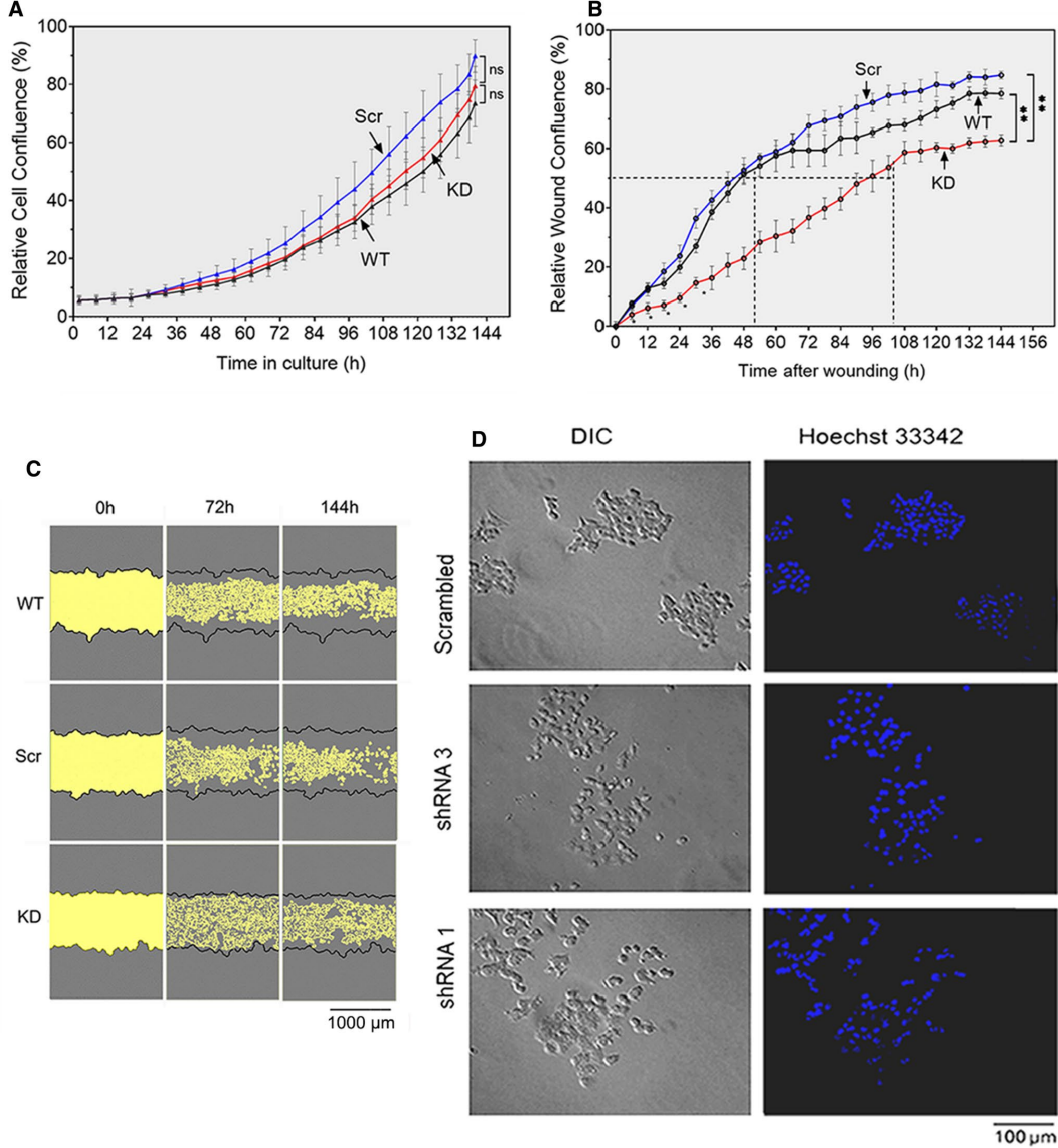
AE2 knockdown modulates SW-48 cell mobility and growth phenotype without affecting proliferation. **A** Cell proliferation rate measurements in AE2 knockdown cells in comparison to wild-type SW-48 cells and knockout controls (SW-48 Scr). IncuCyte^®^ imaging system was used for the experiment. The means (± SD, *n* = 9) of each time point were calculated using the instrument’s software package before plotting against time. Cells stably transfected with the shRNA#3 plasmid were used for the experiment. **B** Cell motility rate measurements using the scratch wound-healing assay. Confluent wells were scratched with an automated pin system, after which wound healing rate was followed against time by measuring cell confluence in the wound area using the in-built software. Cell confluence at each time point is shown as percentages of the wound area. **C** Representative figures are shown for each cell type. Cell-free areas are pinpointed by yellow colour. **D** Growth phenotype of AE2 knockdown cells carrying either the shRNA#1 or the shRNA#3 construct in comparison to control cells (SW-48 Src). In brief, cells were induced for 3 days with doxycycline, then fixed and stained with Hoechst 33,342 DNA dye before imaging with the differential interference contrast (DIC) microscopy. Representative figures are shown for each cell type

To test whether AE2 upregulation has any consequences on SW-48 cell phenotype, we first compared cell proliferation rates between wild type SW-48 (WT), AE2 knockdown cells (KD), and AE2 Scr control cells (i.e., cells that carry the scrambled shRNA construct). AE2 knockdown had no marked effect on cell proliferation rate when compared with either WT or Scr cells (Fig. 5A). Based on a scratch wound healing assay, AE2 knockdown decreased cell migration rate to roughly 50% of that found in wild-type cells (Fig. 5B, C). AE2 knockdown cells also displayed a more dispersed growth phenotype, as the cells did not form similar colonies or cell clumps as did wild-type SW-48 cells (Fig. 5D), suggesting that AE2 knockdown may alter cell–cell contacts or their interaction with the extracellular matrix.

**Fig. 6:**
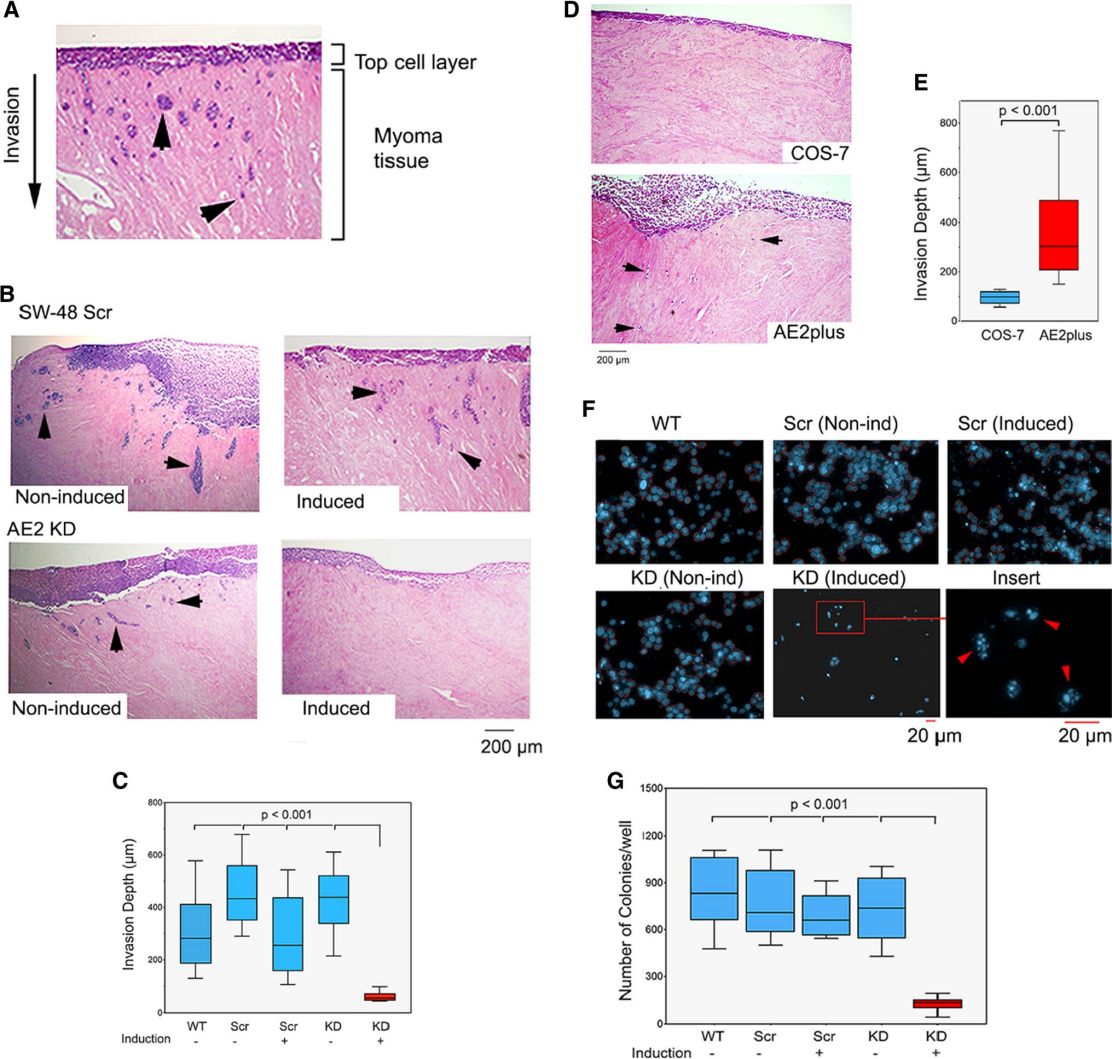
AE2 knockdown in SW-48 cells reverses their invasive and anchorage-independent growth phenotype. **A** Myoma-tissue-based 3D invasion assay. Cells were seeded on top of myoma discs, allowed to grow for 21 days (with or without induction) before fixing and processing for immunocytochemistry. Sections were then cut perpendicularly to the seeded cell layer, stained, and imaged for quantification. Representative figures of invaded cells (invasive foci) inside the myoma tissue are shown (arrowheads). **B** Invasive potential of AE2 knockdown (KD) and control cells (SW-48Scr) were measured as above either with (Induced) or without (non-induced) doxycycline induction. Invasion depth was used as a measure of invasive potential. **C** A box plot presentation showing the median depth of the invasion foci from the top cell layer. Twelve sections from each myoma disc (*n* = 2/24) were used for the quantification with ImageJ software. The whiskers indicate 10th to 90th percentiles. **D** Invasive potential of wild-type COS-7 cells and AE2 overexpressing COS-7 cells (AE2plus). The invasion foci consisted mostly of single cells of which few are marked by arrows to pinpoint their existence. Note their absence in wild-type COS-7 cells. **E** A box plot presentation showing the median invasion depth of the foci in wild type and AE2 overexpressing COS-7 cells. The whiskers indicate 10th to 90th percentiles. **F** Colony formation assay in soft agar. Depicted cells (wild type SW-48cells (WT); AE knockdown cells (KD); SW-48 control cells (Scr) were grown in soft agar for 30 days with or without doxycycline induction (KD and Scr), after which cells were fixed and stained with Hoechst dye before imaging with the Operetta high content screening system. Representative figures are shown. The insert (bottom right) shows a higher magnification of the AE2 knockdown cells with fragmented nuclei (arrowheads). **G** The total number of colonies in each cell type. The number of cell colonies was quantified using the Harmony software. More than three nuclei in a pre-sized area were used to depict one colony. The results are shown as box plots with whiskers denoting 10th and 90th percentiles

An important feature of malignant cells is their invasiveness. Therefore, we assessed whether AE2 upregulation in SW-48 cells is associated with their invasive potential. By using an established 3D human myoma-based invasion assay [44], we found that SW-48 WT cells were highly invasive and showed invasion foci inside the myoma tissue at a 200 to 400 µm distance from the seeded top cell layer (Fig. 6A). Similar foci were also seen with SW-48 Scr cells with or without prior doxycycline induction (Fig. 6B, C). In contrast, no invasion foci were detected induced by AE2 knockdown cells, albeit non-induced cells were invasive. These observations suggested that upregulation of the AE2 protein in SW-48 cells indeed promotes their invasive behaviour. Consistent with this view, we also found that the opposite, i.e., AE2 overexpression in COS-7 cells renders the cell invasive, albeit the foci were small and consisted of only a few cells in each case 6D, E).

Another characteristic feature of cancer cells is their ability to grow and proliferate without attachment to the substratum. Therefore, we employed a colony formation assay in soft agar to test whether AE2 upregulation also contributes to this phenotype. Unlike SW-48 WT cells or non-induced and induced SW-48 Scr control cells, AE2 knockdown cells were able to form colonies only in the absence of doxycycline, but not in its presence (Fig. 6F, G). AE2 knockdown cells were present at low numbers and displayed fragmented nuclei (Fig. 6G insert), suggesting that they represent apoptotic cells. This may reflect the loss of anoikis resistance in AE2 knockdown cells, a special survival program that allows wild-type SW-48 cells to proliferate without any surface contact. Collectively, these findings suggest that upregulation of the AE2 protein in cancer cells has an important role in promoting SW-48 cell malignant phenotype.

### AE2 knockdown attenuates the expression of cancer-associated glycotopes in SW-48 cells

**Fig. 7:**
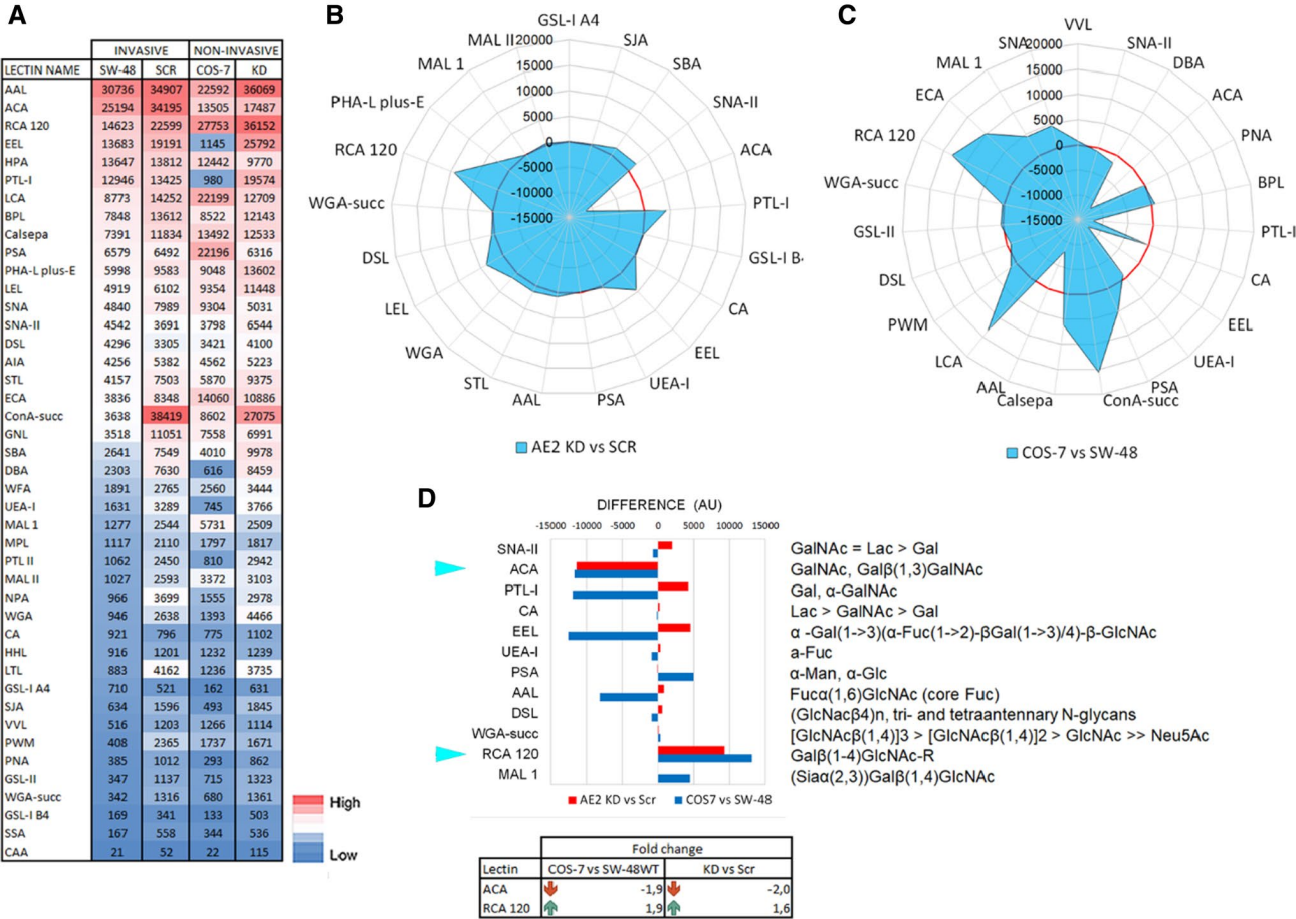
Comparison of glycosylation differences between invasive and non-invasive cancer cells. **A** Heat map analysis showing total lectin binding intensities after sorting them from largest to smallest using the intensities of wild type SW-48 cells as a reference. For each cell type, a heat map was constructed separately using the in-build conditional formatting option in Excel. The means (Fig. S6) were used for calculations. **B** Significant glycosylation differences between AE2 knockdown cells and control cells (KD vs. SW-48 Scr) are shown as a subtracted fingerprint and presented in a radar plot. The calculations were done as above. The red-coloured circle denotes zero values (no difference). **C** A radar plot showing statistically significant (*p* < 0.05) glycan-binding differences between COS-7 cells and SW-48 wild type (WT) cells. **D** Total lectin binding differences (subtracted fingerprints) between non-invasive and invasive cell pairs (AE2 knockdown cells vs. SW-48 Scr and SW-48 WT vs. COS-7). Only statistically significant differences that were common to both cell pairs are shown together with the lectin names and their corresponding glycotopes. Please notice that the overall glycosylation profiles (Fig. S6A, B) of each cell pair are comparable with each other with respect to the most and least abundant glycotopes, justifying their comparison

To uncover why AE2 knockdown in SW-48 cells was able to reverse their invasive and anchorage-independent growth phenotype, we focused on the identification of potential glycosylation changes that differ between non-invasive cells (AE2 KD, COS-7) from the invasive ones (SW-48, SW-48 Scr). The rationale behind this is that cancer-associated glycosylation changes are well known to promote cancer cell invasiveness and metastatic spread [10–12, 45–47]. Moreover, Golgi glycosylation potential is highly dependent on its resting pH as shown above and earlier [13–15, 17, 18]. To accomplish this, we first compared the overall glycosylation patterns (Fig. S6) of the above four cell lines with each other. Heat map analysis (Fig. 7A) revealed that despite cell-type differences, their glycosylation patterns were rather similar, as the most and least abundant glycotopes were detected often with the same lectins. To identify the main differences between non-invasive and invasive cells, we compared AE2 knockdown cells with SW-48 Scr cells (KD vs. Scr) and COS-7 cells with SW-48 wild-type cells (COS-7 vs. SW-48 WT) despite their species difference. The use of SV40-transformed COS-7 cells in the latter cell pair is justified as glycans are highly similar between all mammalian cells [48, 49]. They also continuously proliferate and became invasive upon AE2 overexpression. Comparison of subtracted fingerprint between KD and Scr cells (Fig. 7B) revealed 21 lectins that displayed significantly altered binding of their specific glycotopes. A similar comparison of non-invasive COS-7 cells with invasive SW-48 WT cells revealed significant changes in 23 out of 43 lectins (Fig. 7C). Further comparison of the two cell pairs with each other revealed 12 lectins that were common to both cell pairs (Fig. 7D). Interestingly, of these 12 lectins, only two lectins (ACA and RCA 120) showed comparable changes in both cell pairs. ACA lectin, which is specific for the truncated O-linked Tn- and T-antigens, showed significantly lower (~ twofold) binding in non-invasive cells than in invasive cells. In contrast, RCA 120 lectin that is specific for galactose-containing complex type N-glycans displayed significantly (1.9–1.6-fold) higher binding in non-invasive cells than in invasive cells. Thus, high expression of the truncated O-glycans and low level of terminally galactosylated N-glycans appear to be closely associated with SW-48 cell invasiveness and anchorage-independent growth phenotype.

## Discussion

In this report, we identified the long-sought” proton leakage" pathway that relies on AE2a-mediated bicarbonate-chloride exchange across Golgi membranes. Specifically, we showed that this pathway is strictly dependent on bicarbonate (and Cl^−^) anions as well as the level of the AE2a protein in the Golgi membranes. Therefore, because cytoplasmic bicarbonate and protons are present in the cytoplasm and dictate its resting pH, Golgi resting pH also depends on cytoplasmic pH. Electrochemical calculations also indicated that AE2a-mediated HCO_3_^−^/Cl^−^ exchange is electrochemically favourable and sustained by both proton pumping and chloride channel-mediated Cl^−^ influx down to its concentration gradient. Based on these observations, we presented a model in which proton leak across Golgi membranes involves net acid efflux via the well-known bicarbonate buffering reaction that produces water and carbon dioxide from luminal protons and bicarbonate anions. The reaction (H^+^  + HCO_3_^−^ <  > H_2_CO_3_ <  > CO2 + H_2_O) is reversible and proceeds spontaneously in the acidic Golgi lumen (see the supplementary video S1) while the opposite reaction (the production of H^+^ and HCO_3_^−^) takes place in the cytoplasm and is catalysed by CAII or by other members of this same family. The reaction is utilized universally among living organisms to regulate cytoplasmic and extracellular pH as well as the pH of body fluids [29, 30, 35, 42].

To be physiologically relevant, the two end products must be rapidly expelled across Golgi membranes. We envisage that this occurs either via direct diffusion or aquaporin channels because the latter can transport, besides water molecules, also other small molecules including glycerol, urea, nutrients, metabolic precursors, waste products, toxins, and even gases such as CO_2_ [40, 41]. In this context, it is interesting, and perhaps not even unexpected, to note that the flattened morphology of the Golgi cisternae can accelerate this process as their shape provides an optimal surface-volume ratio for gas and water exchange. The need to prevent water and carbon dioxide from accumulating in the Golgi lumen while keeping Golgi buffering capacity in balance may be one of the main reasons for their unique morphology in mammalian cells. In addition, it is probably not a mere incidence that the pKa of the bicarbonate buffering system is within the same range as the Golgi resting pH in mammalian cells (pKa 6.4 vs. pH 6.7–6.0).

The membrane topology of the AE2 anion exchanger and its pH-dependent anion exchange activity is also consistent with the model. Both the N-and C-termini of the Golgi- and plasma membrane-localized AE2 protein face the cytoplasm and enable various protein interactions that appear to regulate anion exchange activity or the compartmental localization of the protein in the cells [28, 30, 32, 36]. All AE2 isoforms also function against alkaline loads, whereby they export bicarbonate anions (a weak base) to the extracellular space (in exchange for Cl^−^ anions) or the Golgi lumen, given their identical membrane topology. Previously, it has been also shown that the AE2 anion exchange activity is pH-dependent, i.e., it is active above pH 5.0 [50]. Therefore, it retains its activity in the Golgi, where the resting pH is above pH 6.0. Yet, the pH sensitivity of the AE2 protein may act as a safeguard mechanism to prevent other more acidic organelles compartments (such as secretory vesicles or lysosomes) from alkalinizing in a case AE2 might get mislocalized there. Considering this, and the predominant localization of the AE2a variant in the Golgi membranes in a variety of cells, suggests that the AE2-mediated net acid efflux pathway applies only to the Golgi compartment and perhaps also to the endoplasmic reticulum (ER), given that both AE1 and AE2 acquire their anion exchange already in the ER [51].

The functional relevance of the AE2-mediated net acid efflux pathway was verified by showing that both the AE2 mRNA and protein levels are often upregulated in cancers and established cancer cell lines. In SW-48 cells, this could result from the amplification of chromosome 7 (47 + XX, + 7), the chromosome in which the AE2 gene locates in humans [52]. Alternatively, it could be due to epigenetic changes or environmental factors such as hypoxia [13, 53]. Nevertheless, upregulation of the AE2 protein was found to have important consequences on cancer cell phenotype most likely via its ability to modulate Golgi resting pH. We showed that a high AE2 protein level in SW-48 cells was associated with near-neutral Golgi resting pH and that AE2 knockdown restored it to near-normal levels. Moreover, keeping in mind that AE2 protein expression level correlated well with the Golgi resting pH in COS-7 cells (Fig. 1), AE2 overexpression is very likely responsible for their elevated Golgi resting pH. In this regard, it is important to notice that the AE2 protein was expressed also at the cell surface. Therefore, it is possible that AE2 knockdown increases bicarbonate levels (and pH) in the cytoplasm via attenuating their export to the extracellular space, in line with the known functions of the AE2 proteins against alkaline loads. This is in line with our notion that the cytoplasmic pH was slightly elevated in AE2 knockdown cells when compared to control cells. Such an increase, however, did not affect cell proliferation rate but may contribute to Golgi resting pH by counteracting AE2 knockdown-mediated loss of the protein in the Golgi and thus explain why the Golgi resting pH in AE2 knockdown cells plateaued at pH ~ 6.5, i.e., 0.2 pH units above the mean Golgi pH in COS-7 cells.

Recently, Galenkamp et al. [27] introduced a new hypothesis that suggests that the Golgi acts as a proton sink or reservoir in cancer cells. It is well known that cytoplasmic pH is more alkaline in cancer cells than in normal cells and necessary for their proliferation and growth [1–3, 27]. An altered energy metabolism (glycolysis) is needed also for continuous proliferation, however, counteracts this need, as it increases acid load in the cells. Cancer cells can normally cope with this extra acid load by launching several independent proton removal pathways, one of which involves proton import to Golgi lumen via ATP-mediated proton pumping or the sodium-hydrogen exchanger NHE7. Therefore, an increased Golgi buffering capacity in the Golgi (due to AE2 overexpression) seems to be beneficial to cancer cells, as it allows more protons to be neutralized through this pathway. The other side of the coin is that the Golgi resting pH becomes too alkaline for its main tasks including membrane trafficking and glycosylation [13–18]. Such a high buffering capacity in the Golgi may also explain why it has been difficult to demonstrate that the NHE7 functions as an acid loader in the Golgi [25, 26]. However, how other cancer cell types that do not overexpress the AE2 protein, can cope with extra acid load remain unclear but they likely use alternative pathways for expelling protons from the cell for example through the plasma membrane or via endocytic compartments [27]. Another issue that needs to be clarified is why some cancer cell types display elevated Golgi resting pH without AE2 overexpression. We anticipate that in these cells elevated Golgi resting pH likely reflects the altered activity of any of the other transporters or enzymes that contribute to Golgi resting pH. Potential candidate proteins include the V-ATPase, GPHR, GOLAC, CAII, and CAIV. In support of this possibility, our immunoblotting data on CAII showed that this bicarbonate and the proton-producing enzyme are upregulated in MCF-7 cells.

The finding that AE2 knockdown was able to reverse SW-48 cells non-invasive and unable to grow in soft agar without affecting their proliferation markedly is intriguing, as it indicates that AE2 overexpression can promote cancer cell invasiveness and anchorage-independent growth, i.e., two phenomena that are typical for malignant cells. Thus, an important issue is how elevated Golgi resting pH can alter cancer cell malignant behaviour to mimic that of a benign cell. Clarifying this may be challenging due to the multi-faceted nature and the vast number of pathways that contribute to the malignant behaviour of cancer cells. Yet, altered glycosylation may be a unifying factor as it can affect multiple pathways at the same time. Consistent with this, the activity of cell adhesion receptors, receptor tyrosine kinases, death receptors, and matrix metalloproteinases is regulated by their glycosylation status [54–57]. For example, integrins that consist of variable α and β heterodimers, contain multiple potential N-linked glycosylation sites on each subunit. The β1 subunits, which are best characterized, normally carry *N*-acetyllactosamine (Galβ (1,4)GlcNAc) type multi-antennary structures [54]. Their capping with either α(2,3)- or α(2,6)-linked sialic acid and β(1,6)-GlcNac branching was also shown to have profound effects on cell adhesion and motility [56]. In this respect, it is interesting to note that we detected marked differences in the level of this same glycotope between non-invasive (AE2 KD, COS-7) and invasive (SW-48 WT, SW-48 Scr) cells, the former displaying significantly higher levels of this glycotope. Another example of the role of glycans in promoting cancer cell malignant behaviour is the matrix metalloproteinase MMP14, the main enzyme needed for cancer cell invasiveness. Bard and his group [55] recently showed that O-glycosylation of MMP14 by the initial GalNAc residue is markedly enhanced due to relocalization of relevant GALNT glycosyltransferase to the ER. In a mouse liver cancer model, this increase in turn markedly increased MMP14 activity as well as tumour cell invasion and tumour expansion. In addition, impaired O-glycosylation and in particular, the extension of the (sialyl)Tn-antigen to (sialyl)-T-antigen of the TRAIL death receptor was found to alter receptor oligomerization state and to confer apoptosis resistance to the cells [54]. Overexpression of C1GALT1, a key enzyme controlling the elongation of the Tn-antigen to T-antigen in turn was also found to enhance migration, invasion, tumour growth, and metastasis of pancreatic cancer cells [58]. In line with these observations, our lectin microarray analyses showed that the levels of these truncated O-glycan glycotopes were much lower in non-invasive AE2 knockdown cells than in invasive (SW-48 Scr) cells. Taken together, these findings suggest that high expression of the truncated O-glycan glycotopes and low level of terminally galactosylated N-glycans represent potential candidates that may promote cancer cell malignant phenotype. Yet, more detailed glycan analyses, even at the protein- and glycosylation site-specific level [55], are needed to prove or disproof their exact contribution to cancer cell malignant phenotype.

**Fig. 8:**
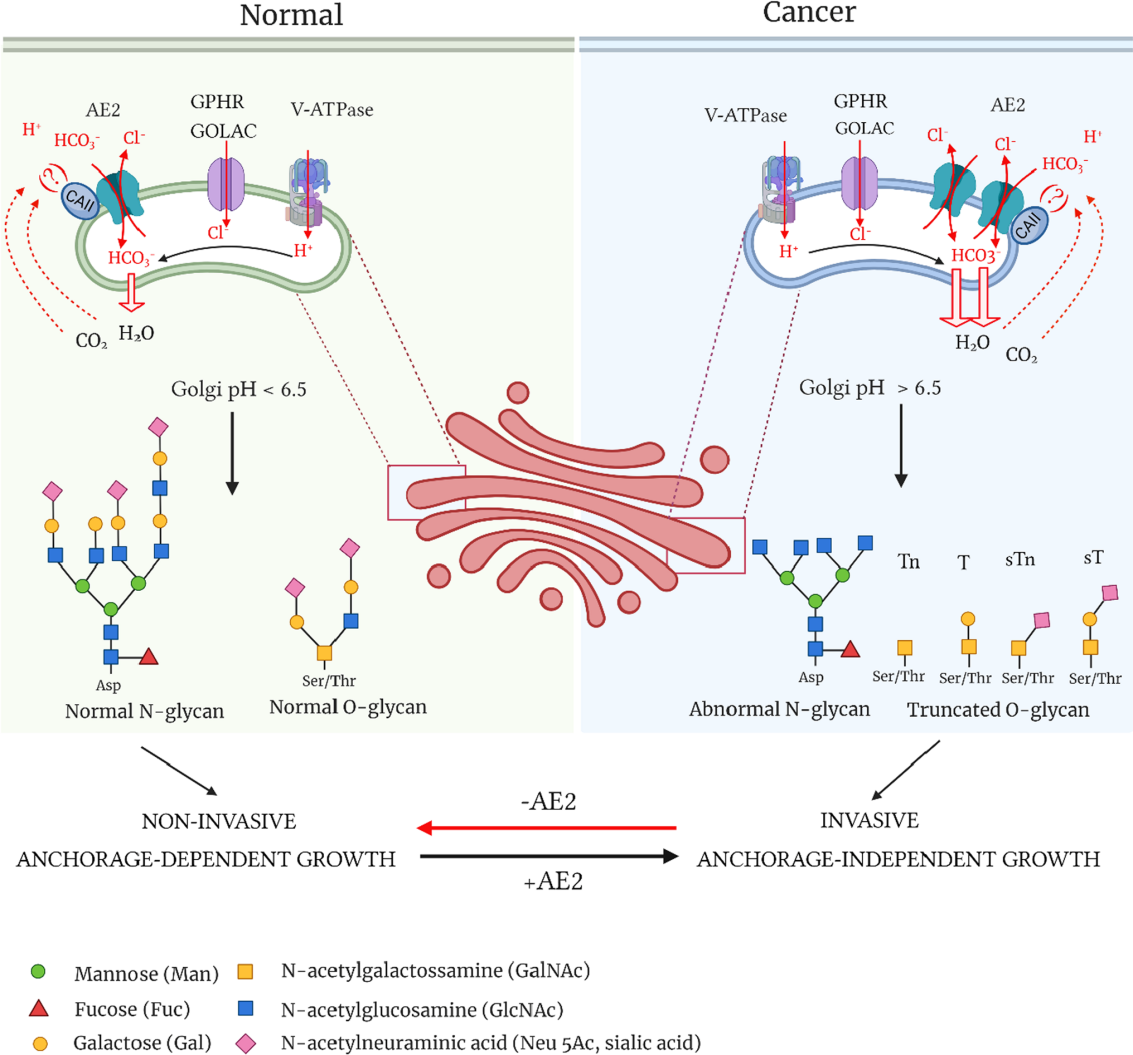
A summary cartoon of the AE2-mediated net acid efflux pathway and its consequences on cancer cell behavior. For more details see the main text

In summary, we showed that overexpression of the AE2 protein in cancer cells promotes their malignant behaviour via enhancing net acid efflux across Golgi membranes (Fig. 8). Thereby, the Golgi resting pH becomes more alkaline which in turn impairs normal processing and maturation of both N- and O-glycan. Altered glycans then promote cancer cell invasiveness and anchorage-independent growth phenotype. We were also able to reverse these phenomena by knocking down the AE2 protein with AE2-specific shRNAs, whereby malignant SW-48 cells lost their invasive and anchorage-independent growth phenotype, i.e. properties typical for benign cells. In support of these data, earlier studies data have shown that the activity of various proteins that regulate cell adhesion, cell motility, invasion, and metastasis is dependent on their glycosylation status. Yet, the exact roles of altered glycans in promoting cancer cell invasiveness and anchorage-independent growth, remain to be clarified. It is also important to emphasize that altered glycans seem to promote these phenomena only in cells that are committed to continuously proliferate and grow (such as SW-48 and SV40-transformed COS-7 cells) since patients with autosomal recessive cutis laxa type 2A (ARCL2A) do not show enhanced cancer risk despite they carry an inactive V-ATPase and display abnormal glycans [59, 60]. Therefore, our findings do not neglect the importance of genetic mutations as the main drivers of tumorigenesis. Rather, they suggest that the main role of altered glycans is to promote tumour progression, i.e., when a benign cell turns into a malignant cell. Thus, our findings may open new possibilities in the future to prevent the metastatic spread of cancers, the main cause of death of cancer patients. In the worst case, they could be exploited to develop new diagnostic tools for detecting metastasis-prone tumours and thereby improve patient care.

## Materials and methods

### Reagents, antibodies, and plasmids

All reagents were purchased from Sigma-Aldrich (St. Louis, MO, USA) unless stated otherwise. The antibodies against the Golgi marker GM130 were purchased from BD Biosciences (#610,822, San Jose, CA, USA). The horseradish peroxidase-conjugated secondary antibodies were from Appliance (Compiègne, France). An antibody against AE2 C-terminal peptide was prepared and affinity-purified as described earlier [61]. Alexa Fluor488- and 594-conjugated anti-mouse and anti-rabbit secondary antibodies and AlexaFluor488- or Alexa594-conjugated peanut agglutinin (PNA) were purchased from Molecular Probes (Eugene, OR, USA) or Invitrogen (Carlsbad, CA, USA). Unconjugated lectins were purchased from either EY laboratories (San Mateo, CA, USA) or Vector Laboratories Inc. (Burlingame, CA, USA). Anti-α-tubulin antibody was from Sigma Aldrich (St. Louis, MO, USA). The plasmid encoding the medial/trans-Golgi localized pHluorin [33] was a kind gift from Dr. G. Miesenböck (Oxford, UK). The AE2-mCherry plasmid was prepared by sub-cloning the full-length AE2 in-frame into the pcDNA3-monomeric Cherry (mCherry) vector (Invitrogen, Carlsbad, CA, USA).

### Cell culture and transfections

All cell lines (COS-7, HeLa, HT-29, SW-48, CaCo-2, DLD-1, MCF-7, MDA-MB 231, RCC4, A431) were from ATCC (Manassas, VA). Cells were grown in high glucose Dulbecco’s modified Eagle’s medium (DMEM) supplemented with Glutamax (Gibco BRL, Grand Island, NY, USA), 10% fetal bovine serum (HyClone, Cramlington, UK), and antibiotics (100 U/ml Penicillin and 100 µg/ml Streptomycin; Sigma-Aldrich, St. Louis, MO) in humidified conditions at + 37 °C and 5% CO_2_. Transfections were performed using either the Lipofectamine® 3000 (Thermo fisher scientific, CA, USA) or the FuGENE 6™ (Promega, Fitchburg, WI, USA) transfection reagents according to the suppliers’ instructions for 1–2 days before the experiments. In certain cases, cells were also transfected with electroporation using Amaxa™ Nucleofector Kit R (Cat.no.VCA-100) and the Nucleofector^TM^II device (program W-001, Lonza Group AG, Cologne, Germany) as suggested by the manufacturer.

### Immunofluorescence electron microscopy

Immunofluorescence microscopy was performed by fixing cells with 2% p-formaldehyde (30 min), after which cells were permeabilized with 0.1% saponin in PBS and stained with the anti-GM130 antibody or with AlexaFluor488- or Alexa594-conjugated lectins (1 h at RT). After washing, cells were treated with relevant species-specific Alexa Fluor488- and 594-conjugated anti-mouse or anti-rabbit secondary antibodies. After mounting, cells were imaged using Olympus BX 51 microscope with appropriate filter sets for the dyes. Alternatively, Zeiss Observer Z1 confocal microscope (LSM 700, Carl Zeiss AG, Oberkochen, Germany) with appropriate filter sets, the Zen2009 software, a 63X Plan-Apo oil-immersion objective, was used. Co-localization studies of the mCherry fusion constructs or GT-pHluorin constructs with the Golgi marker (GM130) antibody were done using Alexa488 or Alexa 594-conjugated secondary antibodies, respectively.

### Immunoblotting

Immunoblotting of the α-tubulin (used as a loading control) was carried out by using sodium dodecyl sulfate–polyacrylamide gel electrophoresis (SDS-PAGE). In brief, cells were lysed on plates with the lysis buffer (50 mM Tris–HCl (pH 7.5), 150 mM NaCl; 1% TX-100; 2 mM EDTA; 2 mM EGTA) supplemented with protease inhibitors (Complete Mini, Roche, Basel, Switzerland). After clearing (15,000*g* for 15 min), 50–100 μg of total protein in SDS-sample buffer was separated using 7.5% SDS-PAGE gels.

Blue-native polyacrylamide gel electrophoresis was used for immunoblotting of the AE2a protein as described below. Cells were washed twice with PBS, scraped from the dishes, pelleted by centrifugation, and lysed on ice in TND/TX-100 buffer (25 mM Tris pH 7.5, 150 mM NaCl, 0.5% Deoxycholic acid, 1% Triton X-100) for 1 h. After clearing by centrifugation (15,000*g*,  + 4C, 15 min), the TX-100 soluble fractions were mixed with 5 × Tris/Glycerol native sample buffer before loading onto a 4–15% Mini-Protean TGX gel (Bio-Rad, Hercules, CA). The samples were run in Tris/Glycine buffer supplemented with 0.02% Coomassie Brilliant BlueG-250 until the samples had migrated 1–2 cm into the gel, and thereafter without the dye at 20 mA for 2 h. The samples were transferred onto the PVDF membrane (Bio-Rad) with Bio-Rad Trans-blot Turbo for a mixed molecular weight program (1.3A-25 V; 7 min). The membrane was quenched by using 5% non-fat milk in TBS-Tween (50 mM Tris-150 mM NaCl-0.02% Tween 20, pH 7.6) supplemented with 0.1% BSA overnight at + 4C. The blot was then incubated with a polyclonal anti-AE2 C-terminal antibody (anti-AE2Ct) or the mouse monoclonal anti α-tubulin antibody (Sigma Aldrich, St. Louis, MO, USA) in TBS-Tween 20 + 0.1% BSA) for 2 h at RT. After washing 3 times for 10 min with TBS-Tween 20, rabbit anti-mouse or goat anti-rabbit HRP-conjugated secondary antibodies (Abliance, Compiègne, France) were added in TBS-Tween-0.1% BSA for 1 h at RT (1:10.000 dilution). After final washings (4 times 15 min in TBS-Tween), protein bands were visualized using the ECL reagent and the GelDoc instrument (both from Bio-Rad, Hercules, USA) before quantification using the build-in Image Lab software.

### Knockdown of AE2a in COS-7 and SW-48 cells

Given the lack of hereditary human diseases related to human AE2, and the severe phenotype described for SLC4A2 (AE2) knockout mice [27], we decided to utilize an inducible shRNA-mediated gene silencing of the AE2a protein (as well as other AE2 transcripts) that also allowed us to control AE2 expression level in the cells. Cells were transfected with the AE2-specific and control (scrambled) shRNAs using the inducible SMART-vector constructs (Dharmacon, Lafayette, CO, USA) as suggested by the manufacturer. The shRNA sequences are shown in Table S1. Stably transfected cells were selected first against puromycin (Gibco, 0.75–1.5 µg/ml, 48 h) before repeated sorting of cells by fluorescent activated cell sorter (BD FACSAria™) and collecting cells with high (> tenfold over the background) doxycycline-inducible RFP expression. Cell lysates were then prepared from the stable transfectants with or without doxycycline induction (50–250 ng/ml) for 72 h and used for the determination of the AE2a protein expression levels by immunoblotting of the cell lysates with the AE2 C-terminal antibody as described above.

### Lectin microarray analyses

Cells cultivated on plates to 70–80% confluency were lysed for 30 min on ice with lysis buffer (50 mM sodium tetraborate buffer, pH 8.5, 150 mM NaCl, 1% Triton X-100) supplemented with the protease inhibitor cocktail (Complete Mini, Roche) and clarified by centrifugation (12,000×*g *at 4 °C for 15 min). 12 μg of total protein of each cell lysate was labelled with 6 μg of NHS activated DyLight 630 dye (Thermo Scientific, Waltham, USA) in 50 μl of labelling buffer (50 mM boric acid/150 mM NaCl, pH 8.5) for 1 h at RT with constant agitation (600 rpm). The reaction was quenched at RT for 1 h by adding 50 µl of quenching buffer (75 mM ethanolamine in 200 mM Tris–HCl/150 mM NaCl, pH 8.5) before dilution (1:12) with the assay buffer (50 mM Tris/300 mM NaCl/2 mM MgCl_2_/2 mM MnCl_2_/2 mM CaCl_2_, pH 7.1) to give the final pH 7.4 to the labelled sample mix. After clearing the mix by centrifugation (12,000×*g* for 10 min at RT), 400 µl of the labeled sample was applied to each well on pre-printed and pre-quenched (with 50 mM ethanolamine) Nexterion H microarray slides (Schott, Germany). The labeling was further incubated in a humidified chamber with constant agitation for 2 h in RT. Slides were then washed five times for 5 min each with the washing buffer (50 mM phosphate buffer/0.05% Tween). Array images were generated using the Genepix 4200AL laser scanner (Axon Instruments) using an appropriate filter set for the DyLight 633™ dye. The mean intensities of bound labels were quantified in triplicate from four parallel arrays (36 measurement spots/sample) using the GenePix Pro^®^ microarray analysis software. Sugar binding specificities of the lectins were deduced from the manufacturer’s product sheets. The corresponding lectin names are available upon request. The specificity of the lectins for their glycotopes was verified during the optimization of the protocol by using labelled fetuin and asialofetuin as markers.

### Organelle resting pH measurements

Golgi resting pH in the cells was determined as follows. Briefly, after equilibration in the assay buffer (PBS supplemented with 0.5 mM MgCl_2_, 0.9 mM CaCl_2,_ and 4.5 g/l d-Glucose (PBS/d-Glucose) at RT without CO_2_, the Golgi region of the cells expressing the GT-pHluorin were imaged by using the Operetta™ high content imaging system (PerkinElmer Inc.). In situ pH calibration was done by replacing the bath solution with different pH calibration buffers (125 mM KCl, 20 mM NaCl, 1.0 mM CaCl_2_, 1.0 mM MgCl_2_) pre-adjusted to pH 7.5, pH 6.5, and pH 5.5 with 20 mM HEPES, MOPS, or MES, respectively. Ionophores (5 μM nigericin, 5 μM monensin) were included in the buffers to dissipate the existing monovalent (H^+^, K^+^, Na^+^) ion gradients. Cytoplasmic pH was measured as above, except that the Golgi targeting signal was omitted from the pHluorin plasmid construct.

Golgi acidification and leakage rate measurements were performed in triplicate as described below. Cells expressing the pHluorin were equilibrated as above, imaged, and then permeabilized with Streptolysin O (SLO, 3 μg/ml) either in chloride-free or chloride-containing high potassium buffer (120 mM KCl, 30 mM NaCl, 10 mM EGTA, 10 mM MgCl_2_, 10 mM Hepes, pH 7.2). Chloride-free high potassium buffer contained corresponding gluconate/sulphate salts instead of chloride anions, which cannot be used for AE2a-mediated bicarbonate exchange [43]. Bicarbonate was added to the chloride-containing bath medium just before the assay was started. Golgi acidification rate (a sum of proton pumping and its leakage rates) was followed by adding fresh ATP (10 mM) to the bath solution. Ratio imaging (15 s intervals) of the selected Golgi regions was performed until the ratios (Golgi pH) reached a plateau. The Zeiss Axio Observer Z1 microscope equipped with a dual FRET camera system and appropriate filter sets for two different excitations (420 and 470 nm) and one emission (500–550 nm) wavelengths were used for imaging. Net acid efflux rates were then measured by replacing the ATP-containing buffer with an ATP-free buffer with added concanamycin A (CMA, 1 µM), a potent V-ATPase inhibitor. Net acid efflux (i.e. Golgi pH increase) was then followed again by ratio imaging until it reached a plateau. At the end of each experiment in situ-pH calibration was performed by using pre-calibrated calibration buffers (pH 5.0, 5.5, 6.0, 6.5, 7.0, and 7.5) as above. Intensity ratios were then transformed to pH values using the determined formulas for the obtained sigmoidal calibration curves. Numerical data were processed using Microsoft® Excel solver (Redmond, WA, USA).

### Immunoblotting of human colorectal cancer tissue samples

All human cancer tissue specimens were obtained from the Pathology research unit, Oulu University Hospital following Institutional Ethical Committee Review Board guidelines (permission numbers. 25/2002, 42/2005, 122/2009). Fresh samples were frozen and stored at  − 80 °C until use. Proteins were extracted using 500 µl of ice-cold lysis buffer (50 mM Tris–HCl, pH 7.4, 150 mM NaCl, 1% Triton X-100, 0.5% deoxycholic acid with a protease inhibitor) and pre-cooled tissue homogenizer (TissueLyser LT, Qiagen GmbH, Hilden, Germany) for 2–4 min at 45 Hz. After homogenization, the lysates were clarified by centrifugation (15,000 rpm, 15 min at + 4 °C) and run on Blue-native gel electrophoresis (for AE2) and SDS–PAGE (for α-tubulin) as described in the main text.

### Cell proliferation and wound healing migration assays

The assays were performed using the IncuCyte^®^ Live-Cell Analysis System and established protocols (Essen BioScience, Newark Close, and UK). In brief, for cell proliferation assays, wild-type SW-48 cells and cells stably transfected with the scrambled or AE2-specific shRNAs plasmids were plated into special 96-well plates (5 × 10^3^ cells/well) and cultured in DMEM supplemented with 10% FCS and penicillin–streptomycin for 6 days. Cells were imaged at 2 h intervals during the 5-day culture period before quantification with the IncuCyte^®^ software.

For the scratch wound cell migration assay, 5 × 10^4^ cells/well were plated and after reaching confluence, cell monolayers were grown on Image Lock 96-well microplates (Essen Bioscience) in DMEM with 1% FCS after scratching 700–800 µm wide “wounds” using the 96 pin IncuCyte^®^ Wound Maker (Essen Bioscience). Wound closure (migration) was then followed by using phase-contrast imaging for 6 days at 2 h intervals. Basic IncuCyte^®^ software and settings were used for the analyses. The data are expressed as percentages of confluence in each well.

### 3D invasion assay

The invasive properties of wild-type SW-48 and AE2 knockdown cells as well as COS-7 cells stably overexpressing the AE2a variant (G418 selection) were investigated using an established organotypic 3D-myoma-invasion model [44]. In brief, myoma discs pre-equilibrated at + 4 °C in DMEM were placed in tightly fitted Transwell® inserts (Corning, Inc., Corning, NY, USA) after which 5 × 10^5^ cells (in 50 μl of DMEM) were added on top of each disc. After attachment, myoma discs with cells were transferred onto uncoated nylon discs placed on curved steel grids (3 × 12 × 15 mm) in 12-well plates, each well-containing 1 ml of fresh media with and without doxycycline. The myoma organotypic cultures were maintained for 14 days with daily media changes. Each assay was performed in triplicate. The specimens were fixed in 4% formalin overnight, dehydrated, and embedded in paraffin. Finally, 6 μm thick sections were cut and deparaffinized before staining with Mayer’s Hematoxylin–Eosin. After imaging, the invasion depth of all invaded cells (area) in each microscopic field was determined by measuring the distance of the cell invasion front from the top cell layer on each disc using the Image J (Fiji) v1.46o (National Institute of Health, USA). Uterine leiomyoma tissues were obtained from routine surgeries of otherwise healthy donors after informed consent. The study was reviewed by the Regional Ethics Committee of the Northern Ostrobothnia Hospital District (license number 2/2017).

### Soft agar colony formation assay

Black wall CellCarrier-96 Ultra Microplates (PerkinElemer, Inc, Waltham, Ma, US) was used in the soft agar colony formation assay. Pre-warmed 25 μl of 2 × Dulbecco’s modified Eagle’s medium (D-MEM; containing 20% FBS, 200 U/ml penicillin, 200 μg/ml streptomycin, and 25 μl of melted 1% Ultra Pure™ agarose (Thermo fisher scientific, CA, USA) solutions were mixed and transferred into each well and put at 4 °C for 30 min to allow the agar layer to solidify. Trypsinized cells (2.5 × 10^3^ cells) were then suspended in 25 μl of D-MEM/10% FBS and mixed with 25 μl of 2 × D-MEM containing 20% FBS and 25 μl of 0.7% agar before adding the suspension on top of the solidified agar bed and cooling at 4 °C for 15 min. After adding 50 μl of 1 × DMEM per well, the plates were incubated for 30 days at 37 °C and 5% CO_2_. The medium supplemented (or not) with 100 ng/ml doxycycline was changed every 2–3 days. Thirty days post-seeding, cells were fixed with 2% PFA at RT for 30 min and stained with the Hoechst 33,342 dye (1 μg/ml) at 37 °C and 5% CO_2_ before imaging with the Operetta™ high content imaging system (PerkinElmer Inc.). Forty fields were acquired from each well using appropriate filter sets, and a 20 × objective. Images were analyzed using the Harmony software with selected scripts to allow segmentation to cell colonies. Segmentation criteria included colony size (> 3 cells/colony), circularity, and fluorescence intensity of nuclei.

### Statistical analyses

Statistical analysis was performed using either Excel or GraphPad Prism Software (GraphPad Software Inc., La Jolla, CA, USA). Unless stated otherwise, the comparison of medians (± SD) between two groups was done by two-tailed Student’s *t* test, whereas multiple groups were compared by one-way ANOVA. All error bars represent standard deviation (SD) and the *p *values < 0.05 were considered statistically significant.

### Supplementary Information

Below is the link to the electronic supplementary material.

Supplementary file1 (DOCX 4095 KB)

Supplementary file2 (MP4 30271 KB)

## Data Availability

The datasets generated during and/or analysed during the current study are available from the corresponding author (elham.khosrowabadi@oulu.fi) on reasonable request.

## Abbreviations

GlcNAc: *N*-acetylglucosamine
GalNAc: *N*-acetylgalactosamine
Gal: Galactose
Man: Mannose
Fuc: Fucose
